# Supplementary material for: Latency reversal plus natural killer cells diminish HIV reservoir in vivo
Source: Nat Commun. 2022 Jan 10;13:121. doi: 10.1038/s41467-021-27647-0 (PMC8748509; doi:10.1038/s41467-021-27647-0)
Supplement: Supplementary file 1 — Supplementary Info [file 41467_2021_27647_MOESM1_ESM.pdf]

Supplementary Information

Latency reversal plus natural killer cells diminish HIV reservoir in vivo

Kim et al.

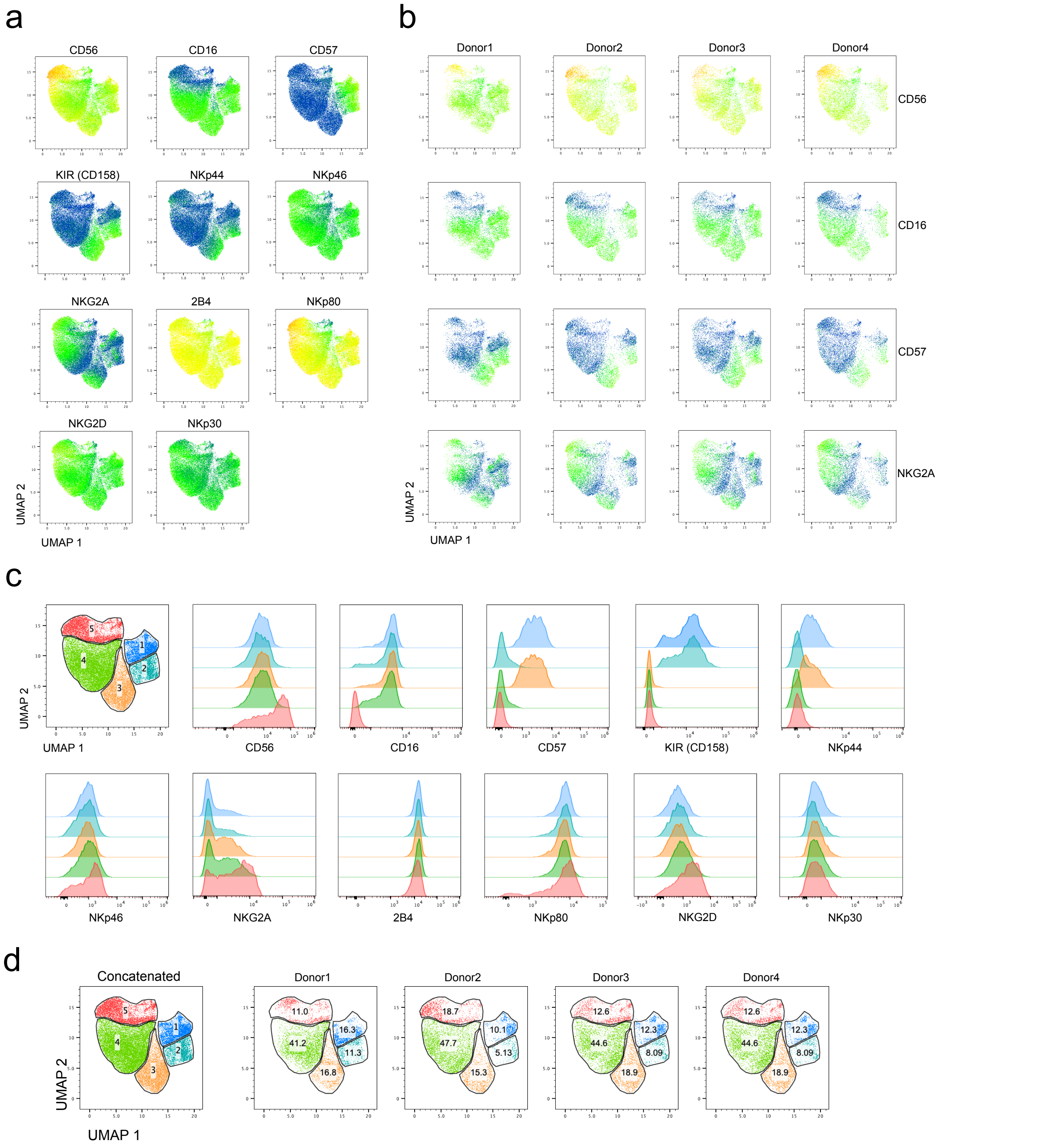

Supplementary Figure 1. UMAP visualization of sorted CD56⁺CD3⁻ cells by flow cytometry. a, b, UMAP analysis of CD56⁺CD3⁻ cells from concatenated (a) and individual donors (b) by flow cytometry. Intensity of the expression of markers was visualized using a rainbow heat scale. c, Manually assigned clusters were assigned on the concatenated sample. Histograms of surface markers are shown with x-axis measuring marker intensity and y-axis normalized to mode. d, Frequencies of manually assigned clusters among CD56⁺CD3⁻ cells across individual donor samples. n=4 biologically independent donors. Results are representative of one independent experiment.

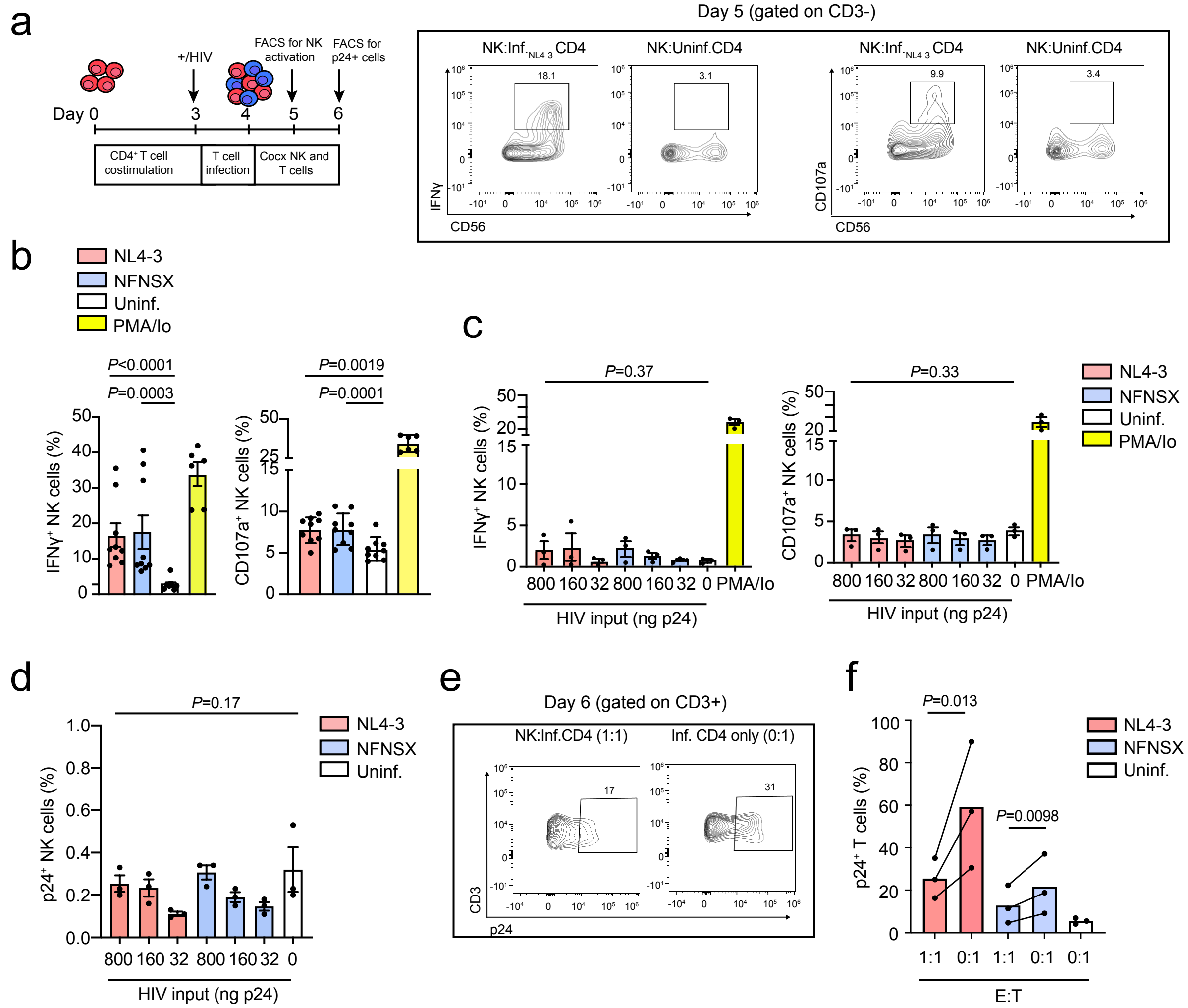

Supplementary Figure 2. NK cells are activated by HIV-infected CD4$^+$ T cells and inhibit HIV replication. a, Experimental design (left) of allogeneic peripheral NK cells and CD4$^+$ T cells. Representative FACS plots of IFN-γ and CD107a expression on the CD3$^-$ gated NK cells when cocultured for 24 h with NL4-3 infected or uninfected CD4$^+$ T cells at a ratio of 1:1. b, Frequency of allogeneic NK cells expressing IFN-γ and CD107a when cocultured with NL4-3 (red) or NFNSX-infected (blue) or uninfected (white) CD4$^+$ T cells at 1:1 ratio for 24 h. Positive control NK cells were activated with PMA and ionomycin (yellow). n=6 independent biologically replicates from three independent experiments. c, d, Frequency of IFN-γ and CD107a (c) or HIV gag p24 (d) expressing NK cells that were exposed to varying doses of cell-free NL4-3 (red) or NFNSX (blue) supernatant or cell media without virus (white) or with PMA and ionomycin (yellow) in the absence of CD4$^+$ T cells. e, f, Representative FACS plots (e) and graph (f) showing the frequency of p24$^+$ CD4$^+$ T cells that were infected with NL4-3 (red) or NFNSX (blue) or uninfected (white) when cultured with or without allogeneic peripheral NK cells at 1:1 ratio for 48 h. n=3 independent biologically replicates from one representative experiment. Shown are mean ± SEM (b, c, d). *P* values were calculated using two-tailed paired t-test (b, f) or one-way ANOVA (c, d). Source data are provided as a Source Data file.

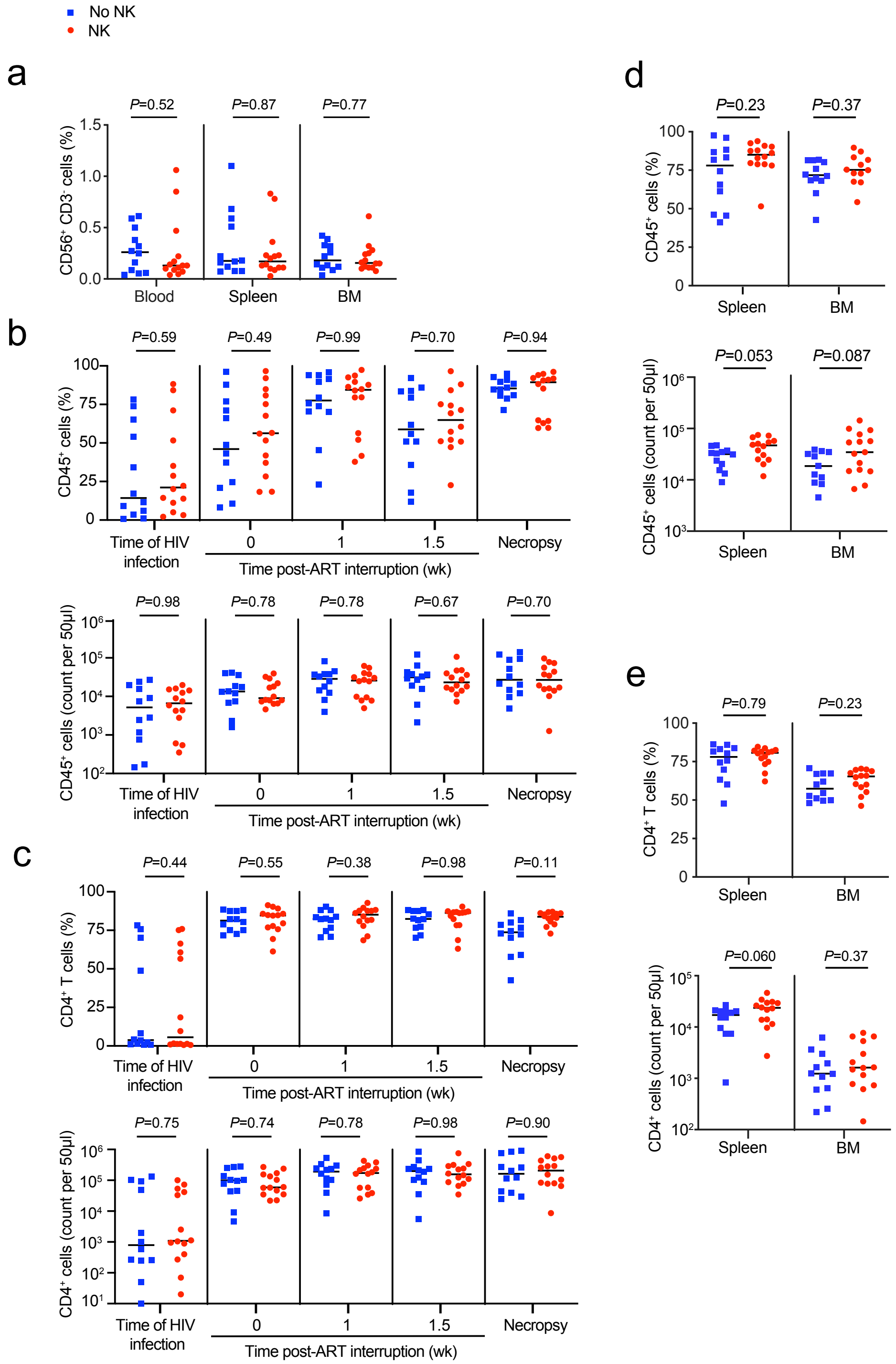

Supplementary Figure 3. Human immune engraftment of NFNSX-infected BLT mice treated with NK cells. a, Frequency of human $CD56^{+}$ $CD3^{-}$ cells in the blood, spleen, and bone marrow "BM" at necropsy in the No NK (blue) and NK (red) groups. b, c, Longitudinal frequencies and absolute counts of human $CD45^{+}$ cells (b) and $CD4^{+}$ T cells (c) in the blood at various timepoints. d, e, Frequencies and absolute counts of human $CD45^{+}$ cells (d) and $CD4^{+}$ T cells (e) in the spleen and BM at necropsy. n=6 biologically independent animals in each group observed over one independent experiment. Horizontal bars represent the medians. *P* values were calculated using two-tailed Mann-Whitney test. Source data are provided as a Source Data file.

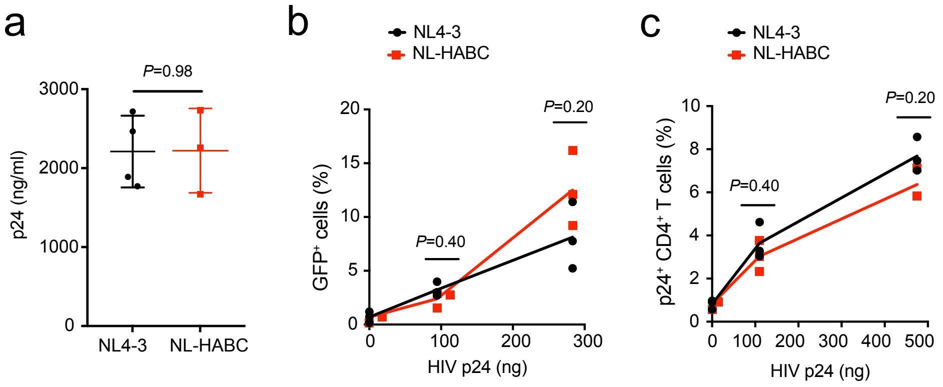

Supplementary Figure 4. Generation and infectivity of barcoded NL-HABC. a, HIV p24 protein levels from the virus supernatant of 293T cells transfected with plasmids encoding NL4-3 (black) or NL-HABC (red). Data is mean ± s.d. n=4 biologically independent transfection preparations for NL4-3 and n=3 biologically independent transfection preparations for NL-HABC. b, c, Varying input of NL43 or NL-HABC was added to GHOST cells (b) or co-stimulated $CD4^+$ T cells (c). Infected cells were quantified 48 h later by flow cytometry. n=3 technical replicates per group (b). n=3 biologically independent donors per group (c). Connecting lines indicate the median. *P* values were calculated using two-tailed unpaired t-test. Data are representative of one independent experiment. Source data are provided as a Source Data file.

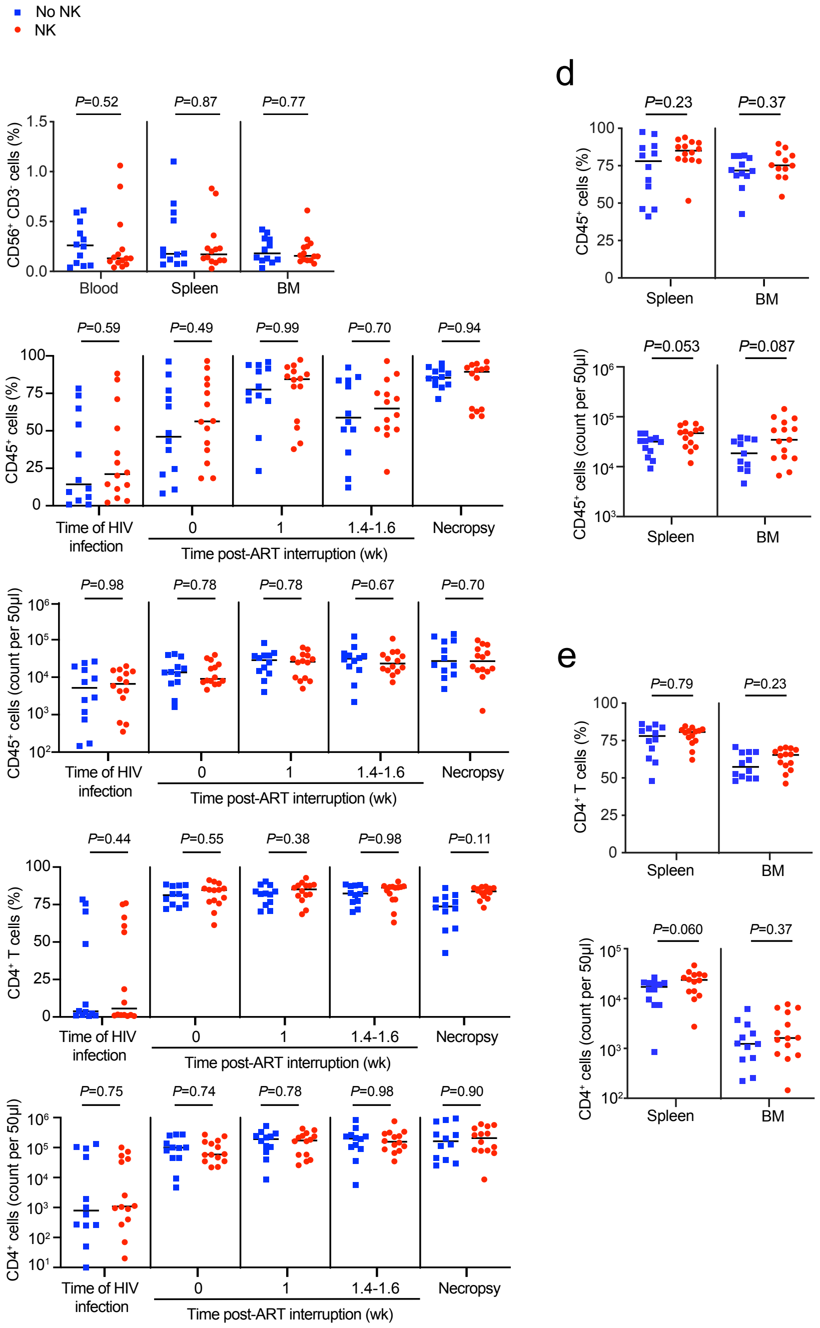

Supplementary Figure 5. Human immune engraftment of NL-HABC-infected BLT mice treated with NK cells. Cells from mice described in the No NK (blue) and NK (red) groups in Fig. 2 were immunophenotyped by flow cytometry. a, Frequency of human CD56$^+$ CD3$^-$ cells in the blood, spleen, and bone marrow "BM" at necropsy. b, c, Longitudinal frequencies and absolute counts of human CD45$^+$ cells (b) and CD4$^+$ T cells (c) in the blood at various timepoints. d, e, Frequencies and absolute counts of human CD45$^+$ cells (d) and CD4$^+$ T cells (e) in the spleen, and BM at necropsy (right). n=12 biologically independent animals in No NK group, n=14 biologically independent animals in NK group Horizontal bars represent the medians. *P* values were calculated using two-tailed Mann-Whitney test. Source data are provided as a Source Data file.

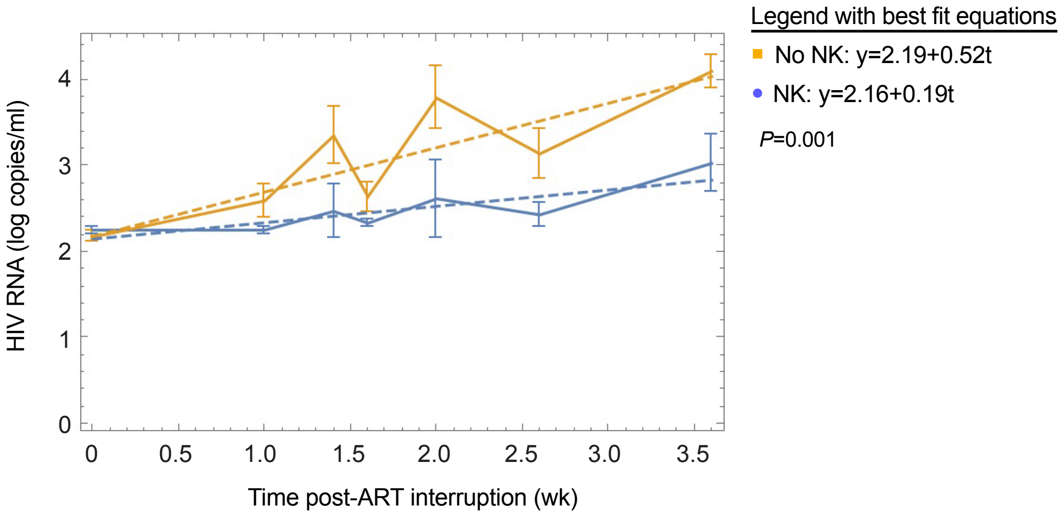

Supplementary Figure 6. NK cells reduce viral spread after ART interruption in NL-HABC infected mice. Viral spread rate in mice that received or did not receive NK cells after ART discontinuation. The mean and the standard deviation of the experimental time-series are shown by solid lines, and linear fit by dashed lines. The best fitting equations for the HIV RNA log copies of virus (y) are provided where time (t) is measured in weeks. Converted to growth rate per day, the values are r=0.181 day^{-1} and r=0.087 day^{-1} for without and with NK cells, respectively. Significance of the difference between the two slopes was calculated by using z-statistics.

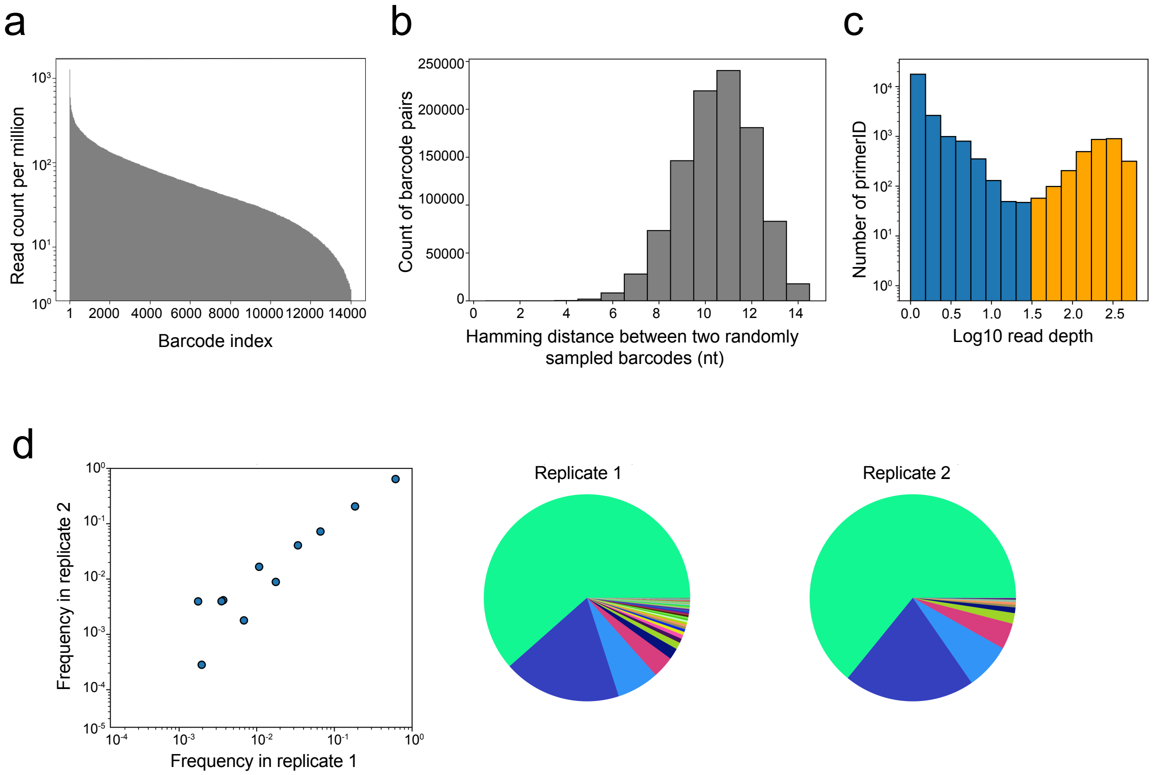

Supplementary Figure 7. Analysis of barcoded NL-HABC. a, Histogram demonstrates ~14,000 viral barcodes with relatively equal distribution as quantified by Hiseq sequencing derived from virion-associated viral RNA of the virus supernatant collected from HIV-producing transfected 293T cells. b, Histogram shows the pair-wise distance between barcodes is 11 bp. c, Representative histogram shows the frequency of Primer ID following a bi-modal distribution of authentic barcodes (orange) versus barcodes containing PCR error (blue). d, RNA molecules from the same population were sampled at two different times to quantify the starting number of RNA molecules in each replicate and thereby assess the reproducibility of the results. Scatterdot plot of the two replicates (c). r =0.92, Spearman coefficient. P=6.7 x 10^{-5}. Pie chart depicting the absolute diversity of clones obtained from the same sample for each replicate (d). Source data are provided as a Source Data file.

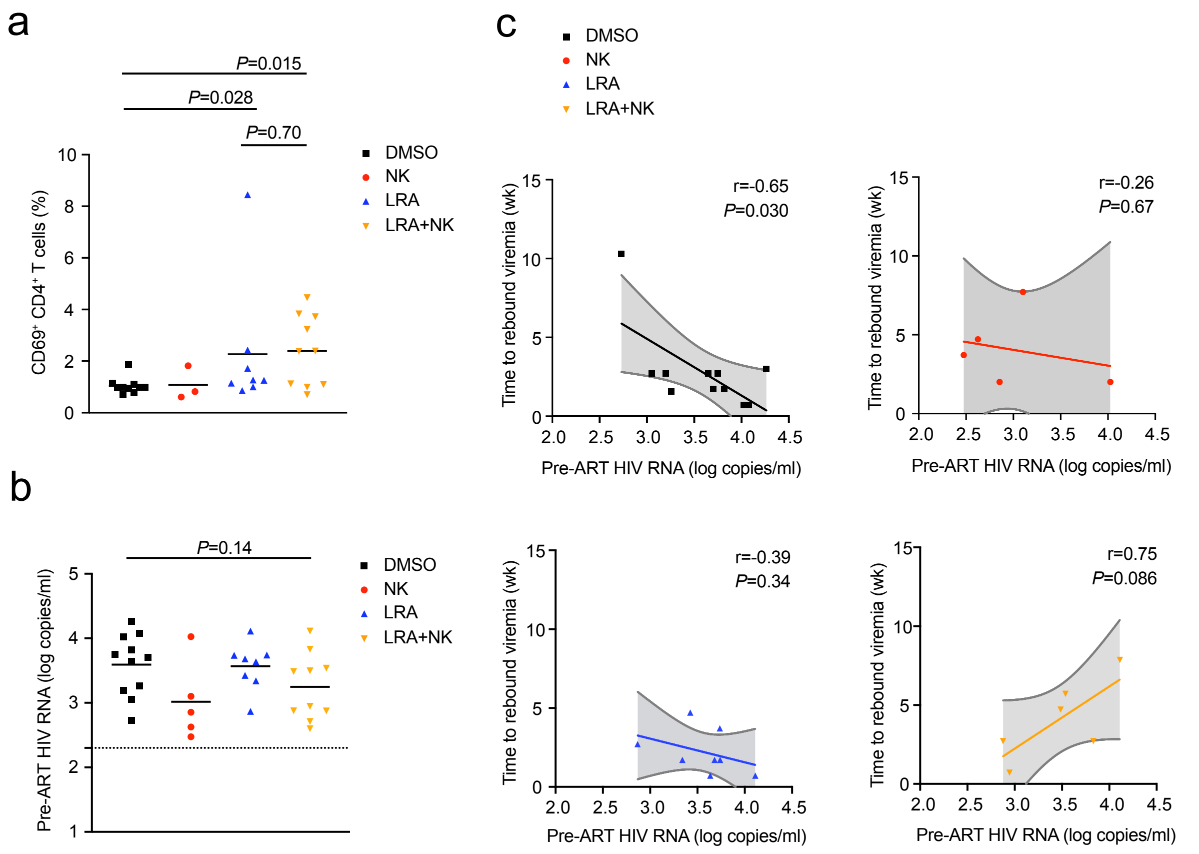

Supplementary Figure 8. T cell activation and correlation between pre-ART viral loads and time to rebound. a, Frequency of $CD69^+$ $CD4^+$ T cells in the blood five days after the injection of DMSO only (black), NK only (red), LRA only (blue), and LRA plus NK (orange) groups. n=10 biologically independent animals in DMSO only group, n=3 biologically independent animals in NK only group, n=8 biologically independent animals in LRA only group, n=10 biologically independent animals in LRA plus NK group. *P* values were calculated using two-tailed Mann-Whitney test. b, Plasma viral loads for each animal before ART was initiated. Black dotted line indicates the detection limit of 2.3 log RNA copies per ml. *P* values were calculated using one-way ANOVA Kruskal-Wallis test. n=11 biologically independent animals in DMSO only group, n=5 biologically independent animals in NK only group, n=8 biologically independent animals in LRA only group, n=10 biologically independent animals in LRA plus NK group. Horizontal bars represent the means (a, b). c, Scatterplot of pre-ART HIV levels and time to rebound after ART discontinuation of the rebounding animals. Among the rebounding animals, n=11 biologically independent animals in DMSO only group, n=5 biologically independent animals in NK only group, n=8 biologically independent animals in LRA only group, n=6 biologically independent animals in LRA plus NK group. Lines are linear predictions of time to rebound on pre-ART viral loads. The 95% confidence intervals of the fitted values are shown by grey areas. r = Pearson correlation coefficient. *P* values were calculated using Pearson correlation test. Results are pooled from two independent experiments. Source data are provided as a Source Data file.

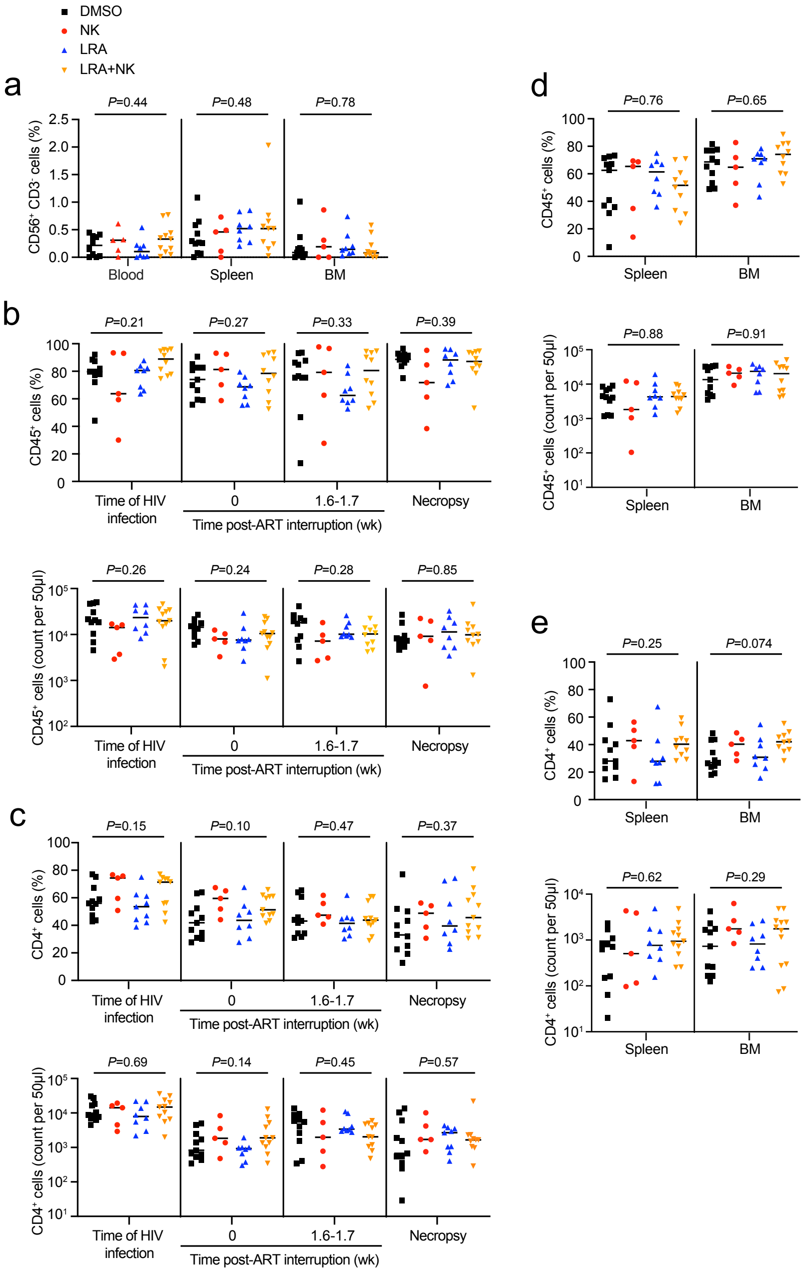

Supplementary Figure 9. Human immune engraftment in infected mice treated with SUW133 and NK cells. a, Frequency of human $CD56^+$ $CD3^-$ cells in the blood, spleen, and bone marrow "BM" at necropsy of the DMSO only (black), NK only (red), LRA only (blue), and LRA plus NK (orange) groups. b, c, Longitudinal frequencies and absolute counts of human $CD45^+$ cells (b) and $CD4^+$ T cells (c) in the blood at various timepoints. d, e, Frequencies and absolute counts of human $CD45^+$ cells (d) and $CD4^+$ T cells (e) in the spleen, and BM at necropsy (right). n=11 biologically independent animals in DMSO only group, n=5 biologically independent animals in NK only group, n=8 biologically independent animals in LRA only group, n=10 biologically independent animals in LRA plus NK group. Horizontal bars represent the medians. *P* values were calculated using one-way ANOVA Kruskal-Wallis test. Results are pooled from two independent experiments. Source data are provided as a Source Data file.

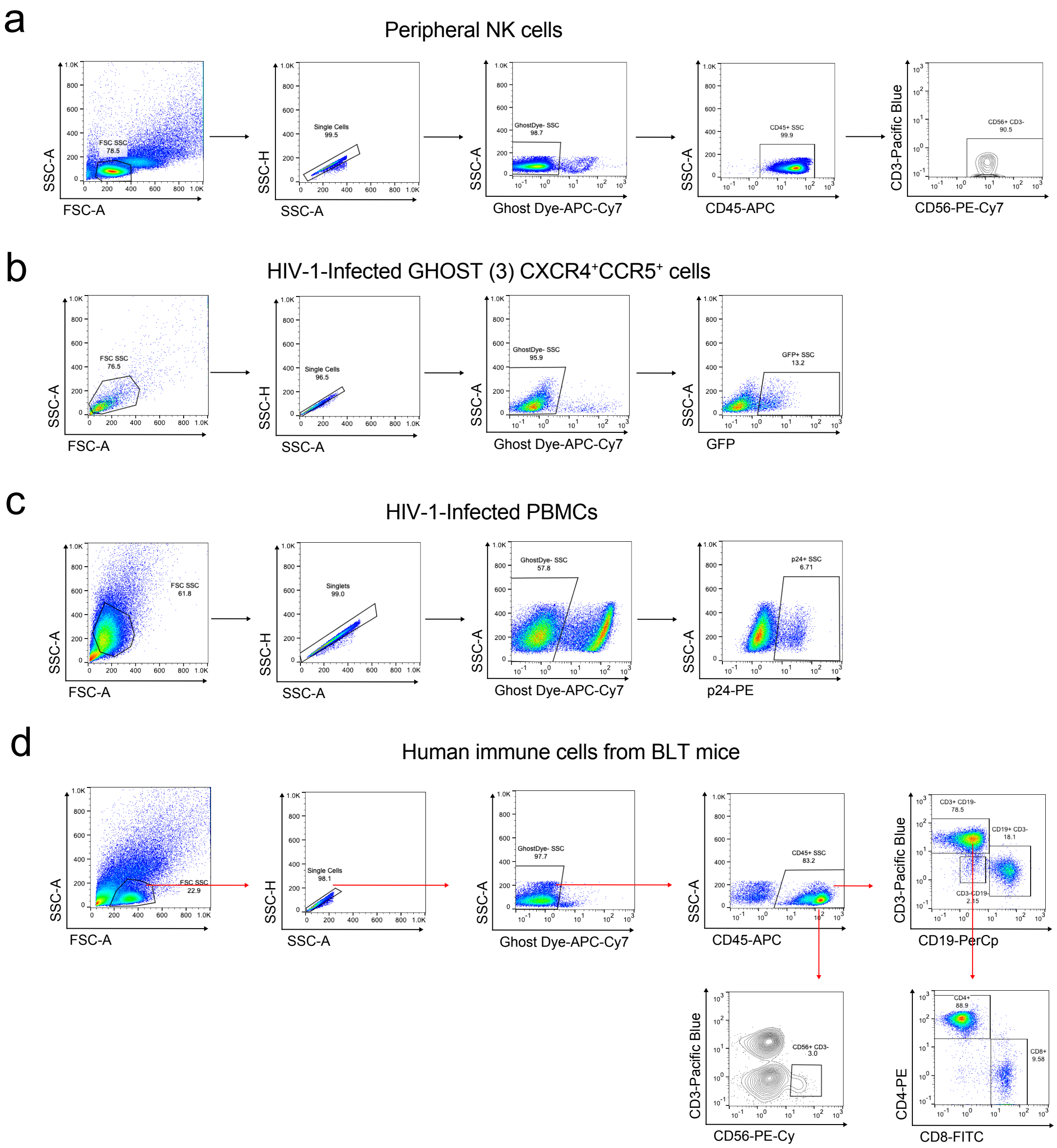

Supplementary Figure 10. Flow cytometry gating strategies. a-d, Flow cytometric analysis of human PBMCs (a), HIV-infected Ghost CXCR4+CCR5+ cells (b), HIV-infected PBMCs (c), and human immune cells from BLT mice (d).

					p24 ng/ml		
Experiment	Mouse ID	Treatment	Rebound viremia?	HIV DNA Spleen?	Day 7	Day 10	Day 14
2	2_1	No NK	Yes	Yes	645.32	280.25	112.76
	2_2	No NK	Yes	Yes	49.52	311.15	133.93
	2_3	No NK	Yes	No	0.84	10.27	241.46
	2_4	No NK	Yes	No	11.51	369.00	773.32
	2_5	NK	No	No	1.30	45.79	85.09
	2_6	NK	No	No	3.09	26.49	36.90
	2_7	NK	Yes	Yes	178.13	197.68	89.89
	2_8	NK	No	No	ND	ND	ND
	2_9	NK	No	Yes	1.03	8.49	273.38
3	3_1	No NK	Yes	Yes	201.50	122.80	48.40
	3_2	No NK	Yes	Yes	1.20	3	ND
	3_3	No NK	Yes	Yes	75.30	134.00	47.00
	3_4	No NK	Yes	Yes	89.90	52.00	18.90
	3_5	No NK	Yes	Yes	568.80	278.10	46.60
	3_6	No NK	Yes	Yes	131.80	66.60	25.20
	3_7	No NK	Yes	Yes	33.50	187.00	258.90
	3_8	No NK	Yes	Yes	49.00	121.70	177.30
	3_9	NK	Yes	Yes	2.30	3.7	ND
	3_10	NK	No	No	2.90	120.20	41.20
	3_11	NK	No	No	ND	ND	165.30
	3_12	NK	Yes	Yes	317.40	180.40	54.10
	3_13	NK	No	No	3.00	104.10	36.80
	3_14	NK	No	No	190.10	65.60	8.60
	3_15	NK	Yes	Yes	15.00	28.10	155.00
	3_16	NK	Yes	Yes	90.60	218.00	264.30
	3_17	NK	Yes	Yes	114.30	102.80	44.10

Supplementary Table 1. Ex vivo viral outgrowth assay of mice that did or did not receive NK cells. Mice from Fig. 2 were tabulated based on their treatment group, presence of rebound viremia after ART interruption and cell-associated HIV DNA in the spleens, and levels of HIV p24 antigen by ELISA of cell-free supernatant from the ex vivo viral outgrowth splenocyte cocultures at 7, 10 and 14 days after coculture. ND = not detected. Lower limit of detection was 0.25 ng of p24 per ml.

Experiment	Treatment	Mouse ID	Time to rebound (wk)	Number of viral barcodes per mouse
3	DMSO	4_1	1.57	3
		4_2	10.3	6
		4_3	2.71	10
		4_4	3	3
		Mean ± s.e.m	4.9 ± 2.7	6.3 ± 2.0
	DMSO+NK	4_5	4.71	4
		4_6	7.71	2
		4_7	3.71	2
		4_8	2	4
		4_9	2	3
		Mean ± s.e.m	4.0 ± 1.1	3.0 ± 0.5
	SUW133+NK	4_10	0.71	4
		4_11	NR	0
		4_12	NR	0
		4_13	NR	0
		4_14	NR	0
		Mean ± s.e.m	8.4 ± 2.8	0.8 ± 0.8
5	DMSO	5_1	1.71	3
		5_2	2.71	3
		5_3	2.71	9
		5_4	2.71	6
		5_5	1.71	4
		5_6	0.71	4
		5_7	0.71	1
		Mean ± s.e.m	1.9 ± 0.3	4.3 ± 1.0
	SUW133	5_8	1.71	11
		5_9	3.71	3
		5_10	0.71	3
		5_11	4.71	3
		5_12	2.71	4
		5_13	0.71	2
		5_14	1.71	4
		5_15	1.71	3
		Mean ± s.e.m	2.2 ± 0.5	4.1 ± 1.0
	SUW133+NK	5_16	5.71	2
		5_17	4.71	3
		5_18	2.71	1
		5_19	2.71	1
		5_20	7.86	1
		Mean ± s.e.m	4.7 ± 1.0	1.6 ± 0.4

Supplementary Table 2. Time to rebound and number of unique barcodes in mice that received SUW133 plus NK cells. Mice from Fig. 5 were tabulated based on their experiment number, treatment group, mouse ID, time to rebound after ART interruption, and number of viral barcodes per mouse. No significant differences in the time to rebound between the DMSO groups in Experiments 4 and 5 (p=0.19, DMSO Exp1 vs DMSO Exp2) or number of barcodes (p=0.66, DMSO Exp4 vs DMSO Exp5). No significant differences in the time to rebound among rebounding animals in the SUW133 plus NK groups in Experiments 4 and 5 based (p=0.39, SUW133 plus NK Exp4 vs SUW133 plus NK Exp5) or number of barcodes (p = 0.14, SUW133 plus NK Exp4 vs SUW133 plus NK Exp5). NR = no rebound. s.e.m = standard error of mean. *P* values were calculated using unpaired two-tailed Mann Whitney t-test.

				p24 ng/ml			
Treatment	Mouse ID	Rebound viremia?	HIV DNA Spleen?	Day 3	Day 7	Day 10	Day 14
LRA+NK	4_11	No	No	ND	ND	ND	ND
LRA+NK	4_12	No	No	ND	ND	ND	ND
LRA+NK	4_13	No	No	ND	ND	ND	ND
LRA+NK	4_14	No	No	ND	ND	ND	ND

Supplementary Table 3. Ex vivo viral outgrowth assay of mice that did or did not rebound after receiving SUW133 plus NK cells. Mice from Fig. 5 that received SUW133 plus NK cells and did not demonstrate presence of rebound viremia after ART interruption and cell-associated HIV DNA in the spleens, were tabulated based on levels of HIV p24 antigen by ELISA detected in the cell-free supernatant from the ex vivo viral outgrowth splenocyte cocultures at 7, 10 and 14 days after coculture. ND = not detected. Lower limit of detection was 0.25 ng of p24 per ml.
