# Supplementary material for: Latency reversal plus natural killer cells diminish HIV reservoir in vivo
Source: Nat Commun. 2022 Jan 10;13:121. doi: 10.1038/s41467-021-27647-0 (PMC8748509; doi:10.1038/s41467-021-27647-0)
Supplement: Supplementary file 3 — Reporting Summary [file 41467_2021_27647_MOESM3_ESM.pdf]

nature portfolio

Corresponding author(s): Jocelyn Kim

Last updated by author(s): Nov 30 2021

Reporting Summary

Nature Portfolio wishes to improve the reproducibility of the work that we publish. This form provides structure for consistency and transparency in reporting. For further information on Nature Portfolio policies, see our Editorial Policies and the Editorial Policy Checklist.

Statistics

For all statistical analyses, confirm that the following items are present in the figure legend, table legend, main text, or Methods section.

n/a	Confirmed	
☐	☒	The exact sample size (n) for each experimental group/condition, given as a discrete number and unit of measurement
☐	☒	A statement on whether measurements were taken from distinct samples or whether the same sample was measured repeatedly
☐	☒	The statistical test(s) used AND whether they are one- or two-sided<br>*Only common tests should be described solely by name; describe more complex techniques in the Methods section.*
☒	☐	A description of all covariates tested
☐	☒	A description of any assumptions or corrections, such as tests of normality and adjustment for multiple comparisons
☐	☒	A full description of the statistical parameters including central tendency (e.g. means) or other basic estimates (e.g. regression coefficient) AND variation (e.g. standard deviation) or associated estimates of uncertainty (e.g. confidence intervals)
☐	☒	For null hypothesis testing, the test statistic (e.g. F, t, r) with confidence intervals, effect sizes, degrees of freedom and P value noted<br>*Give P values as exact values whenever suitable.*
☒	☐	For Bayesian analysis, information on the choice of priors and Markov chain Monte Carlo settings
☒	☐	For hierarchical and complex designs, identification of the appropriate level for tests and full reporting of outcomes
☒	☐	Estimates of effect sizes (e.g. Cohen's d, Pearson's r), indicating how they were calculated

Our web collection on statistics for biologists contains articles on many of the points above.

Software and code

Policy information about availability of computer code

Data collection	For flow cytometric analysis, the data was captured using MACSQuant Analyzer 10 flow cytometer (Miltenyi) and analyzed with FlowJo software (TreeStart, Inc).<br>Viral load data was obtained from CFX96 Touch Real-Time PCR Detection System (Bio-Rad). All qPCR DNA/RNA data were analyzed by GraphPad Prism 7.0 software version (La Jolla, CA, USA).<br>Deep sequencing was performed on HiSeq3000 platform (Illumina) with PE150 settings.
Data analysis	Custom python codes are used to analyze sequencing data using Python 2.7. Flow cytometry analyses were performed using FlowJo v.10, All statistical analyses were performed using Graphpad Prism 8.4.3.

For manuscripts utilizing custom algorithms or software that are central to the research but not yet described in published literature, software must be made available to editors and reviewers. We strongly encourage code deposition in a community repository (e.g. GitHub). See the Nature Portfolio guidelines for submitting code & software for further information.

Data

Policy information about availability of data

All manuscripts must include a data availability statement. This statement should provide the following information, where applicable:

- Accession codes, unique identifiers, or web links for publicly available datasets
- A description of any restrictions on data availability
- For clinical datasets or third party data, please ensure that the statement adheres to our policy

The data supporting the findings in this manuscript are available within the Article and its Supplementary Information. Source data are provided with this manuscript. All raw sequencing reads were deposited on SRA (Short Read Archive) database under the accession number (PRJNA694337). All codes are available on

https://github.com/Tian-hao/BarcodeHIV.

Field-specific reporting

Please select the one below that is the best fit for your research. If you are not sure, read the appropriate sections before making your selection.

- [x] Life sciences
- [ ] Behavioural & social sciences
- [ ] Ecological, evolutionary & environmental sciences

For a reference copy of the document with all sections, see nature.com/documents/nr-reporting-summary-flat.pdf

Life sciences study design

All studies must disclose on these points even when the disclosure is negative.

Sample size	Experimental replicates and group sizes were based on our prior experience with the variance of of the model systems. We selected the sample sizes based on our prior experience and published papers on LRA/drug studies. Thus, sample sizes were determined in order to provide statistical power while also balancing resources (e.g. supply of fetal tissue for humanized mice), costs, and ethical considerations for animal experimentation.
Data exclusions	Only animals with undetectable plasma viral loads at the end of ART treatment and no graft-versus-host disease were included in analysis. This was pre-defined because these experimental factors would confound study of HIV latency and rebound.
Replication	There are four independent experiments reported here in which four series of mice constructed with tissue from 4 different human donors. In addition, we have replicated the kick and kill experiment with one more separate independent series. We confirm all attempts at replication have been successful.
Randomization	Mouse groups were randomized to ensure similar pre-ART viral loads between groups and eliminate the possibility of variations in pre-ART viral load affecting post-ART results.
Blinding	Investigators were not blinded because the investigators wanted to observe if any immediate side effects occurred due to the treatments (e.g. LRA and/or NK cells). However, data and sequence analysis was conducted in a blinded fashion with respect to treatment group.

Reporting for specific materials, systems and methods

We require information from authors about some types of materials, experimental systems and methods used in many studies. Here, indicate whether each material, system or method listed is relevant to your study. If you are not sure if a list item applies to your research, read the appropriate section before selecting a response.

Materials & experimental systems

n/a	Involved in the study	
[ ]	[x]	Antibodies
[ ]	[x]	Eukaryotic cell lines
[x]	[ ]	Palaeontology and archaeology
[ ]	[x]	Animals and other organisms
[ ]	[x]	Human research participants
[x]	[ ]	Clinical data
[x]	[ ]	Dual use research of concern

Methods

n/a	Involved in the study	
[x]	[ ]	ChIP-seq
[ ]	[x]	Flow cytometry
[x]	[ ]	MRI-based neuroimaging

Antibodies

Antibodies used	CD16-Brilliant Violet 785 (clone B73.1, 360733, Biolegend), NKp46-Brilliant Violet 650 (clone 9E2, 331927, Biolegend), NKp30-Brilliant Violet 605 (clone P30-15, 325233, Biolegend), CD69-Brilliant Violet 510 (clone FN50, 310936), CD14-Brilliant Violet 510 (clone M5E2, 301842), CD3-Brilliant Violet 510 (clone OKT3, 317332, Biolegend), CD56-Brilliant Violet 421 (clone HCD56, 362552, Biolegend), CD3-Pacific Blue (clone Hit3a, 310919, Biolegend), CD8-FITC (clone Hit8a, 300906, Biolegend), CD158/KIR-FITC (clone HP-MA4, 339504, Biolegend), CD244-PE (clone C1.7, 329507, Biolegend), CD4-PE (clone RPA-T4, 300539, Biolegend), NKp44 PerCP-Cy5.5 (clone P44-8, 325113, Biolegend), CD19-PerCP-Cy5.5 (clone SJ25, 363016, Biolegend), NKG2D-PE-Dazzle594 (clone 1D11, 320828, Biolegend), NKp80-APC (clone 5D12, 346708, Biolegend), CD45-APC (clone 2Dl, 368512, Biolegend), NKG2A-PE-Cy7 (clone S19004C, 375114, Biolegend), CD107a-PE-Cy7 (clone H4A3, 328618, Biolegend) and CD56-PE-Cy7 (clone MEM-188, 304628, Biolegend); PE-conjugated antibody to HIV core antigen (clone KC57, 6604667, Beckman Coulter).
Validation	We assessed the standard antibody validation that occurs with the vendor at Biolegend and Beckman Coulter. All antibodies are QC tested for human reactivity and are routinely tested for flow cytometric applications (per vendor website). Then, we validate that each antibody on uninfected or HIV-infected human PBMCs and compare to isotype staining. When the antibody is validated, we use it in the real experiment.

Eukaryotic cell lines

Policy information about cell lines

Cell line source(s)	GHOST (3) CXCR4+CCR5+ cells were obtained through the NIH AIDS Reagent Program, Division of AIDS, NIAID, NIH, from Dr. Vineet N. KewalRamani and Dr. Dan R. Littman (cat# 3942).<br>293T cells were obtained from ATCC (ATCC CRL-11268).
Authentication	Obtained directly from NIH AIDS Reagent repository or ATCC. Phenotype and characteristics are as expected therefore they were not subjected to further authentication procedures.
Mycoplasma contamination	All cell lines tested negative for mycoplasma contamination.
Commonly misidentified lines<br>(See ICLAC register)	None used in the study.

Animals and other organisms

Policy information about studies involving animals; ARRIVE guidelines recommended for reporting animal research

Laboratory animals	NOD.Cg-Prkdcscid Il2rgtm1Wjl/SzJ or NSG mice or C57BL/6 Rag2−/−γc−/−CD47−/− or TKO mice were used for humanization. Male and females were used in equal numbers. Mice were between 6-8 weeks old at the beginning of the study. Mice were housed up to five per cage in a 12h light/12h dark cycle at temperatures between 20-26 degrees Celsius and 30-70% humidity.
Wild animals	Not applicable.
Field-collected samples	Not applicable.
Ethics oversight	Animal experiments conformed to all local and national guidelines and regulations (including the Public Health Service Policy on Humane Care and Use of Laboratory Animals, the Guide for the Care and Use of Laboratory Animals, and the AVMA Guidelines for the Euthanasia of Animals), and procedures were approved by the UCLA Animal Research Committee (Approval number ARC-2021-020). Euthanasia was performed by anesthetizing animals with isofluorane followed by cervical dislocation.

Note that full information on the approval of the study protocol must also be provided in the manuscript.

Human research participants

Policy information about studies involving human research participants

Population characteristics	Healthy human donors.
Recruitment	De-identified PBMCs from healthy human donors were obtained by the UCLA AIDS Institute Virology Core Laboratory under IRB approval then provided to investigators in an anonymized fashion.
Ethics oversight	Iinformed consent was obtained by the UCLA AIDS Institute Virology Core Laboratory under IRB approval.

Note that full information on the approval of the study protocol must also be provided in the manuscript.

Flow Cytometry

Plots

Confirm that:

- [x] The axis labels state the marker and fluorochrome used (e.g. CD4-FITC).
- [x] The axis scales are clearly visible. Include numbers along axes only for bottom left plot of group (a 'group' is an analysis of identical markers).
- [x] All plots are contour plots with outliers or pseudocolor plots.
- [x] A numerical value for number of cells or percentage (with statistics) is provided.

Methodology

Sample preparation	Cells are either human peripheral blood mononuclear cells from the UCLA virology core, GHOST (3) CXCR4+CCR5+ cells, which upon HIV infection express GFP upon expression of the viral Tat protein, or single cell suspensions isolated from mouse ttisues. During PBMC cell staining 10E+5 cells were suspended in a 50 µL volume of 1:1 dilution of phosphate buffered saline (PBS):Human AB serum (Sigma). For 1-step staining to detect surface markers, cells were stained with FACS antibodies at 4°C for 20 min and then after washing were resuspended in 3% paraformaldehyde in PBS. Cells were washed in PBS and stored at 4°C prior to running on a MACSQuant Analyzer 10 flow cytomer (Miltenyi). For 2-step staining to detect p24 intracellularly, after surface staining, cells were fixed and permeabilized at room temperature for 30 min. Then stained with p24 antibody at at 4°C for 20 min, and then washed with permeabilization buffer, then cells were washed in permeabilization buffer and resuspended in PBS. Samples were stored at 4°C prior to running on a MACSQuant Analyzer 10 flow cytomer (Miltenyi).

Instrument	MACSQuant Analyzer 10 flow cytomer (Miltenyi).
Software	FlowJo v.10 (TreeStart, Inc)
Cell population abundance	Not applicable.
Gating strategy	Populations of cells were gated based on FSC and SSC. Then doublets were excluded using SSC-A vs SSC-H. Live cells were then gated on based on cells that stained negative for Ghost Dye. Subsequent gating was particular to each experiment. See Supplemental Fig. 10 for further subgating strategy for each cell line and cell type in the manuscript.

☒ Tick this box to confirm that a figure exemplifying the gating strategy is provided in the Supplementary Information.
